# The Role of Dexmedetomidine in Burn Care: Sedation, Analgesia, and Beyond

**DOI:** 10.1093/jbcr/iraf123

**Published:** 2025-08-13

**Authors:** Artur Manasyan, Noah Danesh, Tayla Moshal, Sasha Lasky, Timothy Justin Gillenwater

**Affiliations:** Keck School of Medicine, University of Southern California, Los Angeles, CA 90033, United States; Keck School of Medicine, University of Southern California, Los Angeles, CA 90033, United States; Keck School of Medicine, University of Southern California, Los Angeles, CA 90033, United States; Keck School of Medicine, University of Southern California, Los Angeles, CA 90033, United States; Division of Plastic and Reconstructive Surgery, Keck School of Medicine, Los Angeles, CA 90033, United States

**Keywords:** burn, burn injury, sedation, analgesia

## Abstract

**Background:**

Burn injuries are characterized by intense nociceptive pain, often requiring effective analgesia and sedation during medical interventions and hospital stays.

**Methods:**

We conducted a scoping review to outline the existing literature on dexmedetomidine use in burn patients. Relevant sources were identified through broad searches of five databases (PubMed, Embase, CINAHL, Scopus, and the Cochrane Library), and included materials were reviewed to summarize common themes and reported practices.

**Results:**

Dexmedetomidine, an α2-adrenergic agonist, serves as a promising sedative in this context. Its mechanism of action involves the inhibition of norepinephrine release, thereby modulating pain pathways and inducing a state of sedation without significant respiratory depression. Our review identified that dexmedetomidine is effective not only for ongoing sedation during hospitalization but also for acute short-term sedation during wound dressing changes, which can be particularly challenging for burn patients.

**Conclusion:**

While some studies have noted potential adverse effects, such as respiratory depression and hemodynamic instability, the majority of the literature supports its safety and efficacy in critically ill burn patients. However, there is limited data on its effects on fluid resuscitation, with initial findings indicating a possible need for increased fluid to address hemodynamic changes. Furthermore, research on its impact on wound healing is scarce, emphasizing the need for further studies to better understand its overall role in burn treatment.

## INTRODUCTION

Burns are associated with severe pain that can be a source of significant stress for patients.^1^^,^^2^ Effective analgesia is crucial in the management of pain and in achieving optimal patient outcomes. As such, pharmacological agents commonly used for pain control and sedation in acute burn injury include opioids such as morphine and fentanyl, benzodiazepines such as midazolam, as well as ketamine and propofol for deeper sedation.^3–5^ However, burn injuries trigger systemic inflammatory responses associated with hemodynamic instability, requiring sedatives that can mitigate these effects without compromising respiratory function.^6^^,^^7^

Additionally, standard pain medications may not be as effective for managing burn pain, often necessitating the use of sedation to achieve adequate relief.^8^^,^^9^ Dexmedetomidine (Precedex) is a highly selective α2-adrenergic agonist that offers a unique profile for sedation in burn care. Its mechanism of action involves binding to presynaptic α2-adrenergic receptors in the central nervous system, inhibiting norepinephrine release and subsequently inducing sedation.^10^^,^^11^ Unlike traditional sedatives, dexmedetomidine provides sedation through a non-GABAergic pathway, resulting in a unique profile characterized by preserved respiratory drive and minimal respiratory depression.^10^^,^^11^ This makes it particularly suitable for patients with major burn injuries who require long-term sedation without compromising their already compromised respiratory status. Nevertheless, dexmedetomidine is associated with side effects such as hypotension, bradycardia, which must be carefully monitored.^10^^,^^12^

Previous studies have demonstrated dexmedetomidine’s efficacy in reducing opioid requirements, facilitating earlier extubation in intensive care settings.^13–15^ Its use in burn care represents a promising advancement, offering clinicians a well-tolerated sedative option that aligns with the complex physiological needs of critically ill burn patients. In this study, we aim to summarize the existing evidence on the utility of dexmedetomidine in the context of acute burn care and highlight gaps in the literature to inform future research efforts.

## METHODS

**Figure 1:**
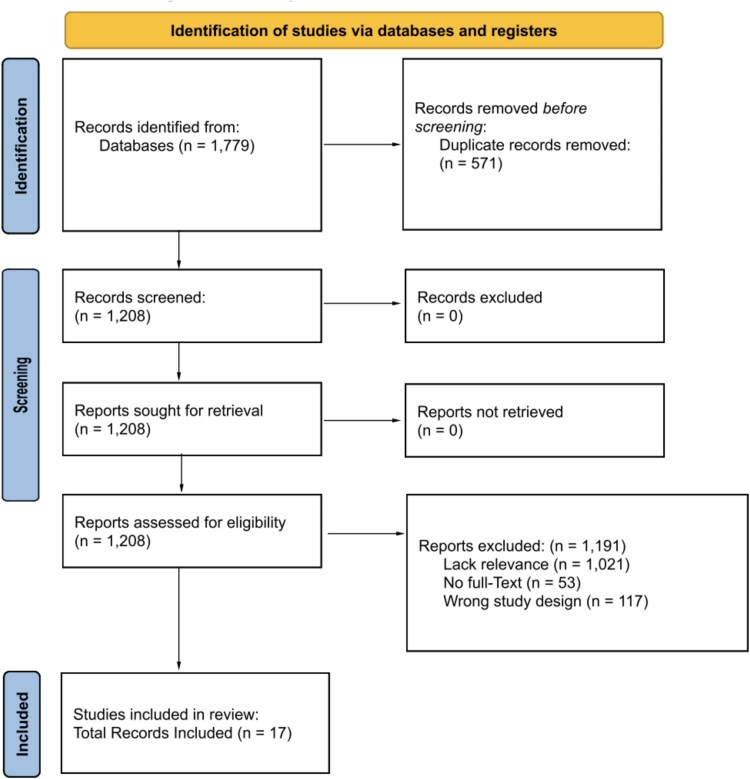
PRISMA Figure of Study Screening

**Figure 2:**
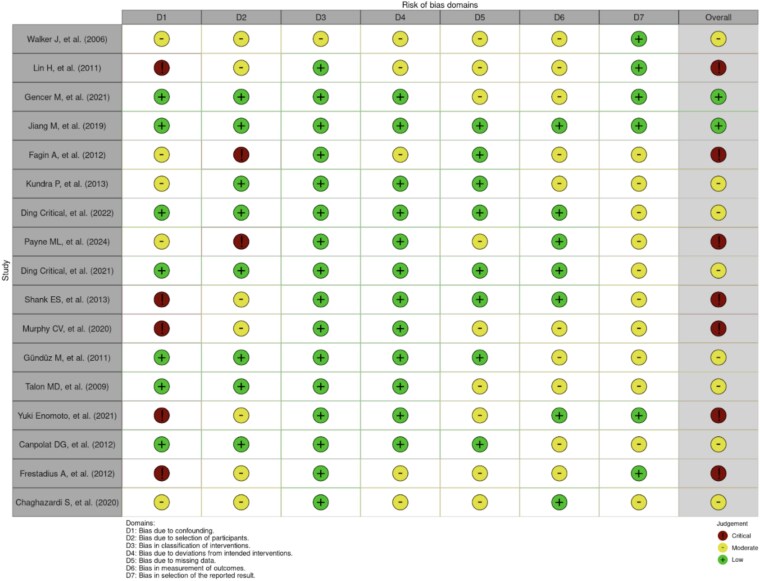
Cochrane Risk of Bias Assessment

The Preferred Reporting Items for Systematic Reviews and Meta-Analysis (PRISMA) guidelines were used to conduct this systematic review. The following databases were queried for relevant studies: *PubMed, Cochrane, Embase, Scopus, Ovid,* and *Web of Science*. Permutations of the following search terms were used in the query: “dexmedetomidine,” “Precedex,” “burn,” “critical care,” “sedation,” “anesthesia,” “analgesia,” and “pain control”. Inclusion criteria were as follows: clinical studies reporting on the safety and efficacy of dexmedetomidine in analgesia, sedation, or other use in patients with acute burn injury. In vitro or animal studies, case reports, letters to the editors, reviews and articles not originally published in English were excluded. Two authors independently screened titles, abstracts, and full texts to determine eligibility for inclusion and exclusion. Disagreements between reviewers were addressed via verbal discussion and a third reviewer. Included studies were assigned a level of evidence (LOE) during data extraction (Table 1). Level I was considered the highest level of evidence, including well-designed randomized controlled trials (RCTs) and meta-analyses, offering robust support for causation. Level II included lesser-quality RCTs and prospective studies, with moderate evidence. Level III consisted of retrospective cohort and case–control studies, with higher bias and limited causal inference. Level IV included case series and observational studies, with significant generalizability limitations. Level V, the lowest level, was based on expert opinions and case reports. Findings from the studies are presented qualitatively, given the heterogeneity in reporting between individual studies. Study inclusion and selection process is illustrated in Figure 1 using the PRISMA flow diagram. Risk of bias assessment was conducted using the Cochrane ROBINS-I (“Risk Of Bias In Non-randomised Studies - of Interventions”) tool, with results summarized in Figure 2.

**Table 1:** Level of Evidence of Included Studies

**Authors (year)**	**Study design**	**Level of evidence (I-V)**
Walker et al. (2006)^23^	Retrospective chart review	Level IV
Lin et al. (2011)^24^	Retrospective chart review	Level IV
Gencer et al. (2021)^18^	Randomized clinical trial	Level II
Jiang et al. (2019)^21^	Double-blinded RCT	Level I
Fagin et al. (2012)^25^	Retrospective review	Level IV
Kundra et al. (2013)^19^	Randomized double-blind cross-over study	Level I
Ding et al. (2022)^20^	Randomized clinical trial	Level I
Payne ML, et al. (2024)	Retrospective cohort study	Level IV
Ding et al. (2021)^22^	Prospective double-blinded study	Level I
Shank et al. (2013)^27^	Prospective preliminary study	Level IV
Murphy et al. (2020)^28^	Open-label, single-arm pilot study	Level IV
Talon et al. (2009)^30^	Randomized prospective double-blind study	Level I
Canpolat et al. (2012)^26^	Randomized prospective study	Level I
Chaghazardi et al. (2020)^29^	Single-blinded randomized comparative study	Level II
Stangaciu et al.^31^	Observational	Level III

## RESULTS

### Use in dressing changes

Dressing changes for burns are often intensely painful due to the removal of adhesive dressings from damaged skin and the disruption of newly formed tissue. Standard pain management approaches may be insufficient because they often do not fully address the depth of the pain, which can involve both surface and deep tissue discomfort, requiring more comprehensive strategies such as as-needed sedation to provide adequate relief.^16^^,^^17^

Dexmedetomidine has proven to be an effective sedative in patients undergoing burn care procedures. In adult burn patients, Gencer et al.^18^ observed in a randomized clinical trial involving 50 patients that dexmedetomidine provided consistent sedation during dressing changes. Patients received a 1 μg/kg dose of dexmedetomidine prior to undergoing the procedure. The authors utilized the Ramsay Sedation Scale (RSS) to measure sedation, a scale in which higher numbers indicate more significant sedation. The study concluded that RSS values during the sedation were higher in the 10 minutes following administration than the baseline RSS values (*P* < .05). Ding et al.^20^ conducted a study comparing dexmedetomidine-butorphanol combination and midazolam-butorphanol combination during dressing changes. Patients were administered a bolus dose of 0.5 μg/kg intravenously over 10 minutes. The authors determined that dexmedetomidine provides similar sedation to midazolam, while also providing an opioid-sparing effect.

Dexmedetomidine offers significant analgesic benefits during dressing changes for patients with burns. Gencer et al.^18^ found that those receiving dexmedetomidine reported lower Visual Analog Scale (VAS) pain scores during and after dressing changes compared to those given midazolam. Jiang et al.^21^ observed similar results in a double-blinded randomized controlled trial with 60 adult patients, where the dexmedetomidine group had significantly lower VAS scores when undergoing surgery than the control group. In addition, Kundra et al.^19^ compared the analgesic efficacy of oral dexmedetomidine (4 mcg/kg) with oral ketamine (5 mg/kg) and demonstrated significant pain relief in burn patients with dexmedetomidine. The mean VAS score was significantly reduced from baseline in both groups (*P* < .05), with Group K (ketamine) showing superior pain relief (overall mean VAS 2.6 ± 0.6 cm) compared to Group D (dexmedetomidine; overall mean VAS 3.8 ± 0.8 cm). It is important to note that the Kundra et al.^19^ study utilized oral dexmedetomidine, which is not currently an FDA-approved modality.

Ding et al.^22^ found that dexmedetomidine provided effective analgesia in their comparative study, which compared butorphanol with dexmedetomidine to butorphanol alone. The study found lower VAS levels for patients using dexmedetomidine in dressing changes, supporting its use in burn care. Dexmedetomidine provides sufficient analgesic effect across different burn patient populations.

### Continuous sedation

Dexmedetomidine has demonstrated effectiveness when used as an ongoing sedative in the critical care of burn patients. Walker et al.,^23^ in a retrospective chart review, found that all 65 pediatric burn patients achieved adequate sedation with intravenous dexmedetomidine, as measured by nursing staff. The duration of DEX infusion ranged from 2 to 50 days, with an average duration of 11 days, and infusions were weaned over 12-24 hours without evidence of rebound hypertension or withdrawal. The patients required sedation over an extended period, particularly following challenges maintaining adequate sedation and analgesia with standard regimens. The average dose of dexmedetomidine provided to the patients was 0.5 μg/kg/hour. Furthermore, Lin et al.^24^ conducted a retrospective chart review that identified patients who received ongoing sedation with dexmedetomidine. Patients were administered a median infusion dose of 0.57 μg/kg/hour without a loading dose, over a median duration of 40 hours. The authors reported a median Riker Sedation-Agitation Score of 3.85 in 11 pediatric patients following administration, indicating moderate sedation. Fagin et al.^25^ compared dexmedetomidine with midazolam in a retrospective review of 42 pediatric patients, finding that dexmedetomidine provided effective ongoing sedation for patients in the intensive care unit. Patients were provided a dexmedetomidine dose of 0.44 μg/kg/hour. While midazolam provided more significant sedation when measured on the RASS, dexmedetomidine was found to provide sufficient sedation with a score of −0.91 ± 0.8. Overall, dexmedetomidine is an effective sedative for burn patients, particularly in the pediatric context.

### Respiratory impact

Jiang et al.,^21^ through a double-blinded randomized controlled trial with 60 adult patients, highlighted dexmedetomidine’s ability to maintain adequate sedation levels without causing significant respiratory depression. Furthermore, a randomized prospective study by Canpolat et al.^26^ that compared the use of ketamine with propofol or dexmedetomidine found the propofol-receiving group experienced significant respiratory depression and hypoxia, whereas patients who were administered dexmedetomidine showed no evidence of respiratory depression and maintained a higher respiratory rate beginning from the fifth minute of the procedure (*P* < .05). Both groups had similar levels of sedation. These results indicate that dexmedetomidine may be a safer alternative for critically ill burn patients who are at risk of respiratory compromise than ketamine with propofol.

### Delirium

Delirium in burn patients is a common complication, often resulting from the compounding effects of severe pain, infection, and medication effects. According to Stangaciu et al.,^31^ the use of dexmedetomidine in burn patients who are being weaned off mechanical ventilation has been shown to reduce the incidence and severity of delirium. Patients in this study received dexmedetomidine 0.5mcg/kg/hour with mean maintenance of 0.72 ± 0.4 mcg/kg/hour. A 1 mcg/kg over 15 minutes as a loading dose, followed by 0.4-0.1 mcg/kg/hour. Dexmedetomidine likely reduces the risk of delirium by promoting sedation with less respiratory depression compared to traditional sedatives, such as midazolam and propofol, and allowing for a more natural sleep pattern. Furthermore, according to Kundra et al.,^19^ dexmedetomidine has been associated with a lower incidence of delirium compared to ketamine, which commonly causes hallucinations and altered mental state. In an analysis of 8 articles by Ng et al.,^32^ dexmedetomidine was shown to significantly reduce the incidence of delirium and agitation, with the delirium incidence reduced to 7.6% compared to 18.5% in the placebo.

### Hemodynamic stability

The studies included in this review highlight mixed results for hemodynamic stability associated with the use of dexmedetomidine in burn patients. Walker et al.,^23^ in a retrospective chart review of 65 pediatric burn patients, found there was 1 instance of hypotension requiring epinephrine administration. The randomized clinical trial by Gencer et al. (2021) compared the efficacy and safety of intravenous dexmedetomidine and midazolam and found no significant differences in hemodynamic parameters—including heart rate, mean arterial pressure, and SpO_2_—between the two groups, suggesting that dexmedetomidine did not significantly adversely impact hemodynamic stability.^18^ Lin et al.^24^ conducted a study on 11 intubated pediatric burn patients and reported no drug-related adverse events, indicating maintained hemodynamic stability throughout the study.

Jiang et al.^21^ conducted a double-blinded randomized controlled trial on 60 adult patients with moderate-to-severe burn injuries. The results demonstrated that the dexmedetomidine group had minimal fluctuations in heart rate and mean arterial pressure. Fagin et al.^25^ performed a retrospective review on 42 pediatric patients with total body surface area burn injuries of 20% or more. The study observed that patients receiving dexmedetomidine had fewer hypotensive episodes compared to those receiving midazolam, indicating better hemodynamic stability with dexmedetomidine.

Some studies indicate dexmedetomidine has an adverse impact on hemodynamic stability. Specifically, in a prospective preliminary study by Shank et al.^27^ on critically injured burn patients, the study authors administered both bolus doses and continuous infusion of dexmedetomidine. The study found that dexmedetomidine administration resulted in a significant drop in MAP, averaging a 27% decrease (SD 7.5%), and an average HR decrease from 146 bpm to 120 bpm (average 19% decrease). Hypotension was observed in all patients post-bolus. In 3 patients, MAP decreased to <50 mm Hg with the bolus dose, and two patients experienced persistent hypotension.

Further, in an open-label, single-arm pilot study by Murphy et al.^28^ with 20 adult burn patients undergoing dressing changes, the results indicated a significant change in heart rate and systolic blood pressure. Specifically, the median pretreatment heart rate was 82 bpm, which decreased to 71 bpm post-treatment. Additionally, the systolic blood pressure showed a reduction, with the median pretreatment SBP 147 mmHg dropping to a median post-treatment SBP of 115 mmHg.

In addition, a prospective, double-blinded study connected by Ding et al.^22^ exploring the use of butorphanol in combination with dexmedetomidine determined that mean blood pressure and heart rate were lower in the combined dexmedetomidine and butorphanol group following administration. Similarly, Chaghazardi et al.^29^ found in a single-blinded randomized comparative study a sudden drop in heart rate and blood pressure following dexmedetomidine administration. Furthermore, in a prospective double blinded study by Talon et al.,^30^ dexmedetomidine was associated with a lower heart rate 5 minutes post-induction compared to patients receiving midazolam (*P* = .01). A study by Canpolat et al.^26^ determined that of 30 patients who received a combination of ketamine and dexmedetomidine, 16.7% experienced bradycardia, 13.3% experienced tachycardia, 6.7% experienced hypotension, and 10.0% experienced hypertension. Ng et al.^32^ further reported an OR value of 2.18 for bradycardia and an OR of 1.89 for hypotension.

Dexmedetomidine generally appears to have the potential to impact different hemodynamic properties, including heart rate and blood pressure, in the immediate term following infusion. Special attention must be given to these parameters following the administration of dexmedetomidine.

### Fluid resuscitation

Payne et al.^33^ evaluated the effect of dexmedetomidine on fluid resuscitation in 170 burn patients, with 115 patients in the control cohort and 55 receiving dexmedetomidine. In this study, patients in the dexmedetomidine cohort received a continuous infusion of dexmedetomidine starting at 0.2 mcg/kg/hour, titrated to achieve a Richmond Agitation-Sedation Scale (RASS) score between −1 and +1, for at least 6 hours within the first 24 hours post-burn injury. The control cohort consisted of patients who did not receive dexmedetomidine and were instead managed with standard sedative and analgesic protocols, as determined by the multidisciplinary team. Payne et al.^33^ found that patients who utilized dexmedetomidine required more fluid in the 24 hours post-burn than control patients (4.2 ± 1.7 mL/kg/%TBSA for dexmedetomidine patients vs. 3.6 ± 1.1 mL/kg/%TBSA for control patients, *P* = .03). There was no significant difference in fluid required at 48 hours post-burn. Dexmedetomidine likely increases fluid resuscitation requirements due to its potential to cause hypotension and bradycardia, necessitating additional fluids to maintain hemodynamic stability. However, this study does not provide sufficient information to determine whether the additional fluid is solely due to hemodynamic effects or if it is at least partially attributable to the volume required for administering dexmedetomidine, which exceeds that of alternative agents. While the evidence on fluid requirements with dexmedetomidine is limited, careful monitoring and adjustment of fluid administration are crucial to balance the sedative effects with hemodynamic stability in this population.

### Considerations

Dexmedetomidine is suitable for patients with acute burn injuries with extensive involvement requiring additional pain management when standard analgesia is not sufficient. It is particularly advantageous for light-to-moderate sedation, such as during procedures or for ventilated patients. Burn professionals should especially consider dexmedetomidine for managing severe acute exacerbation of pain during wound dressing changes, as suggested by our review. The use of dexmedetomidine may be more appropriate in patients with stable hemodynamics and minimal fluid resuscitation needs. Patients receiving dexmedetomidine may require increased fluid resuscitation, though this observation is supported by limited literature, and further research is needed to clarify its impact on fluid management in burn care. In most scenarios, its sedative properties can be utilized effectively without significantly impacting respiratory function.

However, dexmedetomidine should be used with caution in patients exhibiting significant hemodynamic instability or those undergoing extensive fluid resuscitation. Its potential to induce bradycardia and hypotension necessitates rigorous cardiovascular monitoring. For patients with severe cardiovascular compromise or high fluid requirements, alternative sedative options may be preferable to mitigate risks associated with dexmedetomidine. Additionally, ongoing assessment of sedation depth and mental status is critical, as dexmedetomidine may mask or contribute to delirium. Continuous monitoring of fluid balance and hemodynamic status is essential to ensure patient safety and the effectiveness of sedation.

### Future directions

Future research on dexmedetomidine in burn care should explore its effects on wound healing, such as elucidating whether dexmedetomidine’s anti-inflammatory properties and modulation of sympathetic activity can enhance healing rates and reduce complications like hypertrophic scarring. Additionally, optimizing dosing strategies to balance sedative effects with hemodynamic stability will be crucial, particularly in critically ill burn patients who require tailored anesthesia and sedation protocols. Further clinical trials are needed to establish clear guidelines for dexmedetomidine use in burn care, ensuring it maximizes therapeutic benefits while minimizing risks associated with prolonged sedation and potential fluid management challenges.

## CONCLUSION

Dexmedetomidine demonstrates promising outcomes in burn care, particularly in providing effective analgesia and sedation, supporting its role in managing these aspects of burn treatment. However, the literature lacks sufficient data regarding its impact on fluid resuscitation strategies, although preliminary findings suggest a potential need for increased fluid administration to counteract hemodynamic changes. Furthermore, research concerning dexmedetomidine’s influence on wound healing is lacking, highlighting the necessity for further investigation to elucidate its comprehensive effects in burn care settings.

## Notes

Author contributions: Artur Manasyan (Conceptualization [lead], Data curation [lead], Formal analysis [lead], Writing—original draft [lead], Writing—review & editing [lead]), Noah Danesh (Conceptualization [equal], Data curation [equal], Writing—original draft [equal], Writing—review & editing [equal]), Tayla Moshal (Conceptualization [equal], Data curation [equal], Writing—review & editing [equal]), Sasha Lasky (Conceptualization [equal], Data curation [equal], Writing—original draft [equal]), and Timothy Justin Gillenwater (Conceptualization [lead], Data curation [lead], Formal analysis [lead], Writing—review & editing [lead])

Funding: None declared.
